# Biodegradable Nanoparticles of mPEG-PLGA-PLL Triblock Copolymers as Novel Non-Viral Vectors for Improving siRNA Delivery and Gene Silencing

**DOI:** 10.3390/ijms13010516

**Published:** 2012-01-04

**Authors:** Jing Du, Ying Sun, Qiu-Sheng Shi, Pei-Feng Liu, Ming-Jie Zhu, Chun-Hui Wang, Lian-Fang Du, You-Rong Duan

**Affiliations:** 1 Department of Ultrasound, Shanghai First People’s Hospital Affiliated to Shanghai Jiaotong University School of Medicine, 85 Wu Jin Road, Shanghai 200080, China; E-Mails: beautydujing@163.com (J.D.); sqs630904@yahoo.com.cn (Q.-S.S.); 2 Cancer Institute of Renji Hospital Affiliated to Shanghai Jiaotong University School of Medicine, 2200 Xie Tu Road, Shanghai 200032, China; E-Mails: ysun@shsci.org (Y.S.); liupeifeng@yahoo.cn (P.-F.L.); zhumingjie438@hotmail.com (M.-J.Z.); 3 Sino-American United Biotechnology Institute Co., Ltd, 15 Zhong Jiang Road, Shanghai 200032, China; E-Mail: qican@163.com

**Keywords:** gene silencing, nanoparticles, PLGA, PLL, siRNA delivery, GFP

## Abstract

Degradation of mRNA by RNA interference is one of the most powerful and specific mechanisms for gene silencing. However, insufficient cellular uptake and poor stability have limited its usefulness. Here, we report efficient delivery of siRNA via the use of biodegradable nanoparticles (NPs) made from monomethoxypoly(ethylene glycol)-poly(lactic-*co*-glycolic acid)-poly-l-lysine (mPEG-PLGA-PLL) triblock copolymers. Various physicochemical properties of mPEG-PLGA-PLL NPs, including morphology, size, surface charge, siRNA encapsulation efficiency, and *in vitro* release profile of siRNA from NPs, were characterized by scanning electron microscope, particle size and zeta potential analyzer, and high performance liquid chromatography. The levels of siRNA uptake and targeted gene inhibition were detected in human lung cancer SPC-A1-GFP cells stably expressing green fluorescent protein. Examination of the cultured SPC-A1-GFP cells with fluorescent microscope and flow cytometry showed NPs loading Cy3-labeled siRNA had much higher intracellular siRNA delivery efficiencies than siRNA alone and Lipofectamine-siRNA complexes. The gene silencing efficiency of mPEG-PLGA-PLL NPs was higher than that of commercially available transfecting agent Lipofectamine while showing no cytotoxicity. Thus, the current study demonstrates that biodegradable NPs of mPEG-PLGA-PLL triblock copolymers can be potentially applied as novel non-viral vectors for improving siRNA delivery and gene silencing.

## 1. Introduction

Small interfering RNA (siRNA) is a promising biological strategy for treatment of diverse diseases, because of their superior ability to silence target genes in a specific manner. It has been demonstrated that siRNA induces sequence-specific degradation of complementary mRNA, leading to knock down of a target protein in post-transcriptional level [1,2]. Nevertheless, the use of siRNA in clinical applications has been questioned due to several obstacles including poor intracellular uptake and severe enzymatic degradation under *in vivo* circumstances [3,4]. To overcome the limitations, various cationic polymers, peptides, and lipids have been extensively utilized to form nanosized polyelectrolyte complexes via electrostatic interactions with siRNA [5–7]. These polyelectrolyte complexes could protect siRNA from degradation by nucleases and facilitate cellular uptake of siRNA into target cells or tissues by an endocytic pathway [8−10]. Nowadays, nanoparticle-mediated delivery of biomolecules has attracted much attention of the researchers in areas related to therapeutics. It is also expected that due to smaller size, nanoparticles (NPs) will be less susceptible to reticuloendothelial system clearance and will have better penetration into tissues and cells, when used for *in vivo* therapy. However, there are still serious problems such as cytotoxicities induced by cationic carriers and stability in the presence of serum [11]. Much effort, therefore, has been dedicated to the development of efficient carrier materials that are non-toxic, biocompatible and biodegradable.

Polymeric NPs prepared from biocompatible and biodegradable poly(lactic-co-glycolic acid) (PLGA) have been extensively investigated as non-viral gene delivery systems due to their favorable physicochemical characteristics in terms of the safety and achieving sustained release [12]. A major drawback of PLGA NPs is that they are hydrophobic and have a high negative charge on their surface, which limits the interaction with the negatively charged DNA/RNA, in addition to the poor transport characteristics of the DNA/RNA encapsulated PLGA NPs through the cell membrane [13]. As a result, such a system, when administered in experimental animals, is rapidly opsonized by the defense system of the body (Reticuloendothelial System, RES or Mononuclear Phagocyte System, MPS). Incorporation of additional excipients such as polyethylene glycol (PEG) used to coat the PLGA NPs has been attempted as a method to improve the solubility of the NPs, minimize their aggregation caused by hydrophobic surfaces, endow these nanoparticles with ‘stealth’, or RES/MPS evading properties, and finally, improve transfection efficiency [14,15]. Previous studies indicate that nanoparticles fabricated using PLGA alone result in poor encapsulation of siRNA due to the negative charges on their surface [16,17]. The cationic polymer, poly(l-lysine) (PLL) has been widely applied in gene delivery vectors. The primary ɛ-amine groups of lysine in PLL could electrostatically interact with negatively charged DNA or siRNA [18,19] and help to improve the affinity to proteins and cells [20]. In addition, due to the large number of active functional groups with amino, PLL could be modified with various kinds of ligands to achieve active targeting to tissues and cells [21]. On the basis of PEG-PLGA, in order to improve the siRNA loading capacity, here we try to design a biodegradable triblock copolymer monomethoxypoly(ethylene glycol)-poly(lactic-*co*-glycolic acid)-poly-l-lysine (mPEG-PLGA-PLL), which combines the physicochemical characteristics of cationic polymer PLL, biodegradable polymer PLGA and PEG.

Based on these considerations, the purpose of the present work was to synthesize the novel biodegradable mPEG-PLGA-PLL NPs encapsulating siRNA, and examine the physical properties of NPs and *in vitro* siRNA release profile from NPs. The extent of intracellular siRNA uptake and GFP gene silencing effect were evaluated to explore the potential of mPEG-PLGA-PLL NPs as non-viral vectors for gene transport.

## 2. Results and Discussion

### 2.1. Characterization of mPEG-PLGA-PLL Triblock Copolymer

A novel mPEG-PLGA-PLL triblock copolymer was obtained by acidolysis of mPEG-PLGA-PZLL that was synthesized from the ROP of Nɛ-CBZ-l-lysine NCA with amino-terminated mPEG-PLGA-NH_2_ as a macroinitiator. The results measured by the GPC method showed that mPEG-PLGA-PLL triblock copolymer had a molecular weight of 11 kDa.

**Figure 1:**
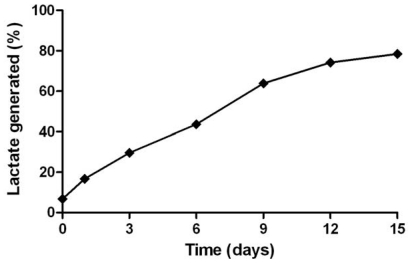
Lactate generation from mPEG-PLGA-PLL with incubation time in PBS (pH 7.4).

The basic characteristic of mPEG-PLGA-PLL triblock copolymer involved in the degradation study was presented in Figure 1. The final *in vitro* degradation products of mPEG-PLGA-PLL were oligomers of lactic acid, oligomers of glycolic acid, lactic acid, glycolic acid, mPEG and PLL. The degradation of mPEG-PLGA-PLL was evaluated by measuring lactate generation with incubation time in PBS (pH 7.4). With prolonged incubation time, the rate of lactate formation gradually increased, and degradation rate of triblock copolymer approached 80% on 15th day.

### 2.2. Physicochemical Characterization of NPs Loading siRNA

**Figure 2:**
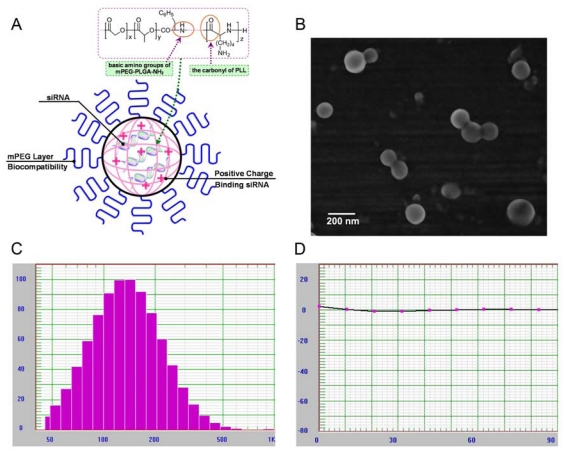
Characterization of NPs loading siRNA. (**A**) Schematic illustration of NPs loading Cy3-siRNA in PLGA-PLL core with mPEG arms; (**B**) SEM photograph of mPEG-PLGA-PLL NPs containing Cy3-siRNA; (**C**, **D**) Size distribution and zeta potential of NPs loading siRNA measured with a particle size and zeta potential analyzer.

**Figure 3:**
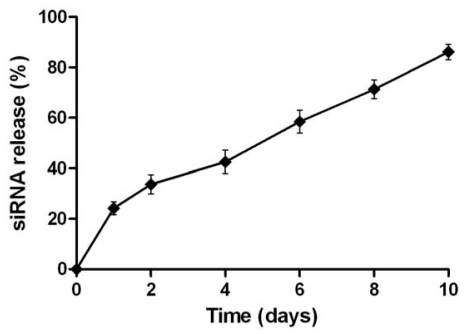
*In vitro* release profile of siRNA from NPs.

We adapted a w/o/w double emulsion method to fabricate Cy3-siRNA loaded NPs composed of mPEG-PLGA-PLL triblock copolymers. Schematic illustration of NPs loading siRNA in PLGA-PLL core with mPEG arms was shown in (Figure 2A). The cationic PLL tightly bound siRNA in the inner water phase. Furthermore, mPEG-PLGA-PLL NPs formed a rigid structure due to ionic interaction between the basic amino groups of amino-terminated mPEG-PLGA and the carboxylic anions of PLL. The presence of mPEG chains in the outer shell layer was used to improve the stability and biocompatibility of NPs. SEM photograph confirmed that the mPEG-PLGA-PLL NPs had a spherical structure (Figure 2B). The mean diameter of blank NPs was 140 ± 62 nm and the zeta potential was +11.4 mV. The size of NPs binding siRNA was 151 ± 74 nm (Figure 2C), whereas zeta potential of NPs loading siRNA was found to decrease to −0.13 mV (Figure 2D). HPLC was used to determine the Cy3-siRNA encapsulation efficiency in NPs and the release profile of Cy3-siRNA from NPs *in vitro*. The encapsulation efficiency of siRNA in mPEG-PLGA-PLL NPs was 86.06%. Figure 3 showed the release profile of siRNA from NPs in PBS (pH 7.4) at 37 °C. Biodegradable NPs prepared from mPEG-PLGA-PLL triblock copolymers sustained the longer-lasting siRNA release over 10 days after the initial burst of 20–30%.

### 2.3. Gel Retardation Assay

**Figure 4:**
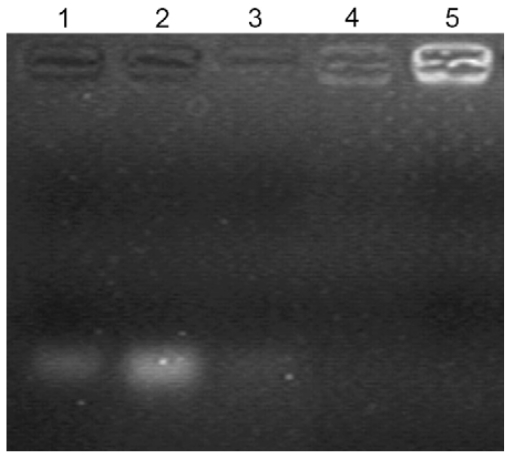
Gel retardation assay of mPEG-PLGA-PLL/siRNA nanocomplexes. Lane 1, a 21 bp siRNA marker; lane 2, siRNA alone; lane 3, the triblock copolymer/siRNA weight ratio of 300:1; lane 4, a weight ratio of 600:1; lane 5, a weight ratio of 900:1.

The preparation of mPEG-PLGA-PLL/siRNA nanocomplexes could lead to the electro-neutralization of the negative charge of siRNA, which were unable to migrate under the influence of electric field during gel electrophoresis. To confirm the formation of the triblock copolymer/siRNA nanocomplexes by the combination of mPEG-PLGA-PLL with siRNA, a gel retardation assay was performed in this study. As shown in Figure 4, complete binding of siRNA with the mPEG-PLGA-PLL (without the presence of a free siRNA band) could be observed when the weight ratio of the triblock copolymer/siRNA was at 600:1, which was consistent with our preparation method of NPs loaded with siRNA. Furthermore, the retardation of migration was more obvious when the triblock copolymer/siRNA weight ratio was increased. However, when the weight ratio of the triblock copolymer/siRNA was at 300:1, a weak free siRNA band in lane 3 could be observed, indicating insufficient complexation of siRNA with copolymer.

### 2.4. Celluar Uptake and Location of NPs Loading siRNA

**Figure 5:**
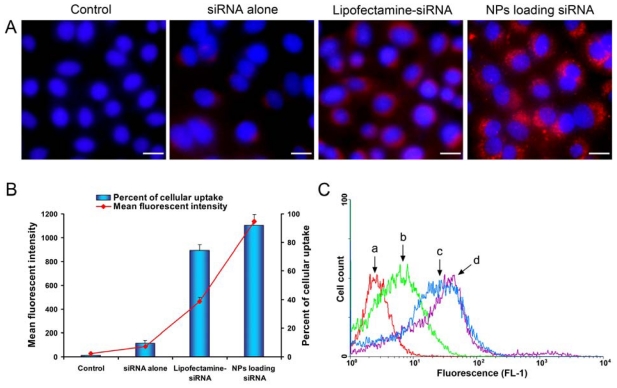
Intracellular uptake of siRNA. (**A**) Fluorescent images of cellular uptake of siRNA in the control and different treatment groups. Red, Cy3-labeled siRNA; blue, nuclei stained with Hoechst 33258. Scale bars represent 20 μm; (**B**) Quantitative analysis of cellular uptake of siRNA using FACSCalibur; (**C**) The flow cytometric picture of control cells (a, red) and SPC-A1-GFP cells treated with siRNA alone (b, green), Lipofectamine-siRNA (c, blue) or NPs loading siRNA (d, purple) were shown.

**Figure 6:**
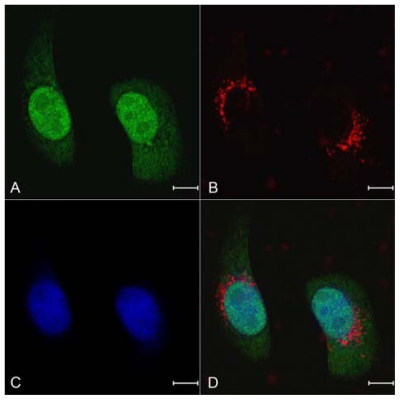
Localization of intracellular NPs loading Cy3-labeled siRNA. SPC-A1-GFP cells were treated with NPs loading Cy3-labeled siRNA and observed at 1000× magnification by a confocal microscope for (**A**) green fluorescence; (**B**) Cy3 red fluorescence; or (**C**) blue fluorescence of nuclei stained with Hoechst 33258; (**D**) A merged image of (**A**), (**B**) and (**C**) was shown. Scale bars represent 10 μm.

We compared the cellular uptake efficiencies of siRNA alone, Lipofectamine-siRNA complexes or NPs loading siRNA. In order to visualize the uptake, siRNA was fluorescently labeled with Cy3 conjugated at the 5' end. After 12 h of incubation, examination of the cultured SPC-A1-GFP cells with fluorescent microscope showed NPs loading siRNA had much higher intracellular siRNA delivery efficiencies than siRNA alone and Lipofectamine-siRNA (Figure 5A). Quantitative cellular uptake of Cy3-labeled siRNA was estimated using a FACSCalibur flow cytometer, which further confirmed the result observed by fluorescent microscope (Figure 5B and 5C). The maximal siRNA uptake efficiency was obtained in the NPs group, which was significantly higher than that of the Lipofectamine-siRNA group (92.17 ± 2.54% *vs.* 74.63 ± 3.84%). However, there was only a small amount of uptake detected in cells treated with siRNA alone. Examination of mean fluorescent intensity of the cultured SPC-A1-GFP cells with flow cytometry showed a similar trend to the percentage of Cy3-siRNA positive cells in the same treatment group. We clearly noticed intracellular localization of the siRNAs was in the perinuclear cytoplasmic region (Figure 5A), and the accumulation of granular NPs loading siRNA was also seen in the perinuclear cytoplasm (Figure 6).

### 2.5. *In Vitro* Gene Silencing

**Figure 7:**
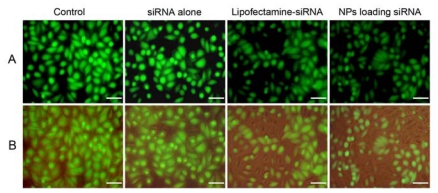
Fluorescent microphotos of SPC-A1-GFP cells treated with various siRNA formulations after 48 h of transfection. (**A**) The green fluorescent protein expression was observed under fluorescent microscope; (**B**) Merged images of the green fluorescent protein and cells seen under light microscope were shown. Scale bars represent 50 μm.

Gene knockdown efficiency of mPEG-PLGA-PLL NPs loading siRNA was estimated by carrying out transfections in stable GFP expressing SPC-A1 cells and compared to the commercially available transfection reagent, Lipofectamine™ 2000. The efficacy of NPs for gene silencing was determined by using FACS measurement and RT-PCR analysis. The qualitative analysis of level of gene silencing was carried out by observing under an inverted microscope after 48 h of transfection. The fluorescence microscopy images showed that the treatment with NPs loading siRNA effectively suppressed the expression of GFP in the cells, compared to other samples (Figure 7).

**Figure 8:**
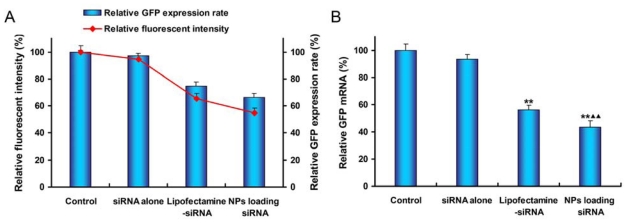
Comparison of gene silencing efficiency of various siRNA formulations after 48 h of transfection. (**A**) The level of GFP expression was estimated by the flow cytometric quantitation of green fluorescence; (**B**) RT-PCR analysis for relative GFP mRNA level was shown. (** *P* < 0.01 *vs.* control group, ^▴ ▴^ *P* < 0.01 *vs.* Lipofectamine-siRNA group).

Quantitative analysis of GFP silencing efficiency of various siRNA formulations was measured by using a FACSCalibur flow cytometer. The relative level of GFP expression in cells treated with siRNA transfection was standardized by setting GFP expression rate and mean fluorescent intensity of control cells to 100% (Figure 8A). After 48 h of transfection, mPEG-PLGA-PLL NPs showed 33.71% inhibition of gene expression, which was higher than that for Lipofectamine-siRNA (25.42% inhibition). There was statistical difference between the two groups (*P =* 0.004). Examination of mean fluorescence intensity of the cultured SPC-A1-GFP cells with flow cytometry demonstrated the green fluorescence signal was decreased by 45.31% when cells treated with NPs loading siRNA while that for Lipofectamine-siRNA was 34.49% (*P =* 0.007). Both GFP expression rate and fluorescent intensity of cells treated with siRNA alone showed no difference as compared to control cells (*P* > 0.05).

To further establish the gene knockdown efficiency at the mRNA level, a RT-PCR analysis of SPC-A1-GFP cells was carried out. The results showed that NPs loading anti-GFP siRNA had a great inhibitory effect to GFP gene expression than the other samples (Figure 8B). The inhibition effect of siRNA-loaded nanoparticles in SPC-A1-GFP cells was 56.33% while that for Lipofectamine-siRNA was 43.72% (*P =* 0.006). However, naked siRNA revealed negligible silencing effect.

### 2.6. Cell Viability Assay

**Figure 9:**
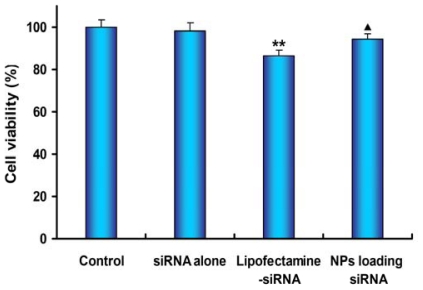
Cell viability assay. SPC-A1-GFP cells were treated with various siRNA formulations under the transfection conditions. The assays were done in triplicate and the standard error was shown. (** *P* < 0.01 *vs.* control group, ^▴^ *P* < 0.05 *vs.* Lipofectamine-siRNA group).

To estimate the extent of toxicity caused by various siRNA formulations, MTT colorimetric assay was performed 48 h after transfection (Figure 9). SPC-A1-GFP cells were treated with various siRNA formulations as described in transfection and gene silencing experiment. The treatment of cells with mPEG-PLGA-PLL NPs loading siRNA did not induce obvious cell toxicity. However, cell viability decreased to 86.39 ± 2.84% after 48 h of Lipofectamine-siRNA treatment, which was significantly lower than those of the NPs group and the control group (*P =* 0.018 and *P =* 0.001, respectively). These results further confirmed that mPEG-PLGA-PLL NPs loading siRNA had the advantage with lower toxicity than Lipofectamine™ 2000.

### 2.7. Discussion

Small interfering RNA has been widely investigated as a potential therapeutic for treatment of various diseases. However, the use of siRNA is limited due to its rapid degradation and insufficient cellular uptake *in vitro* and *in vivo*. Despite the high transduction efficiency of viral vectors which are derived from viruses by the use of recombinant DNA techniques, their clinical potential should be fully understood in terms of issues related to production, safety and immune response that need to be addressed [22]. The non-viral vectors have recently gained increasing attention due to their stability, safety, ease of preparation, and are easily manufactured for large-scale production for treatment of numerous acquired or inherited human diseases [23,24]. Generally, nonviral vectors include liposomes, cationic polymers, and nanoparticles. Even though liposomes have been developed that are capable of delivering DNA or RNA through the cellular membrane, and achieving high activity of the RNA interference, they have shown a relatively low encapsulation efficiency of siRNA molecules, poor storage stability, and rapid clearance from the blood [25,26]. The formation of complexes from cationic polymers with anionic DNA or oligonucleotide solutions was applied by many research groups [27–29]. The simplicity of these self-assembling polyelectrolyte complexes is both an advantage and a drawback. Although such complexes are easy to generate and can protect DNA from enzymatic degradation, they are always characterized by a broad size distribution and variable shape [29], and most of these cationic polymers are not readily biodegradable, which induces high toxicity and necrosis [30–32].

Biodegradable polymeric NPs based on PLGA have been widely investigated as carriers for DNA and oligonucleotides due to their small particle size, favorable safety profile and sustained-release characteristics [33]. With regards to formulation development, it is very challenging to efficiently encapsulate high amounts of hydrophilic macromolecular compounds like siRNA into uniform, nano-sized PLGA particles, mainly due to the hydrophobic nature of the PLGA and the absence of electrostatic interactions between siRNA and PLGA [34]. In our present study, a novel triblock copolymer mPEG-PLGA-PLL was obtained by acidolysis of mPEG-PLGA-PZLL that was synthesized from the ROP of Nɛ-CBZ-l-lysine NCA with amino-terminated mPEG-PLGA-NH_2_ as a macroinitiator. The triblock copolymers mPEG-PLGA-PLL could combine the characters of cationic polymer PLL, biodegradable polymer PLGA and PEG: siRNA-loaded NPs fabricated using triblock copolymers possessed a PEG loop structure to increase the stability and biocompatibility, hydrophobic PLGA segments as the core, and the primary ɛ-amine groups of lysine in PLL to electrostatically interact with negatively charged phosphate groups of siRNA to deposit with the PLGA core. As a result, siRNA was incorporated into the mPEG-PLGA-PLL NPs with an encapsulation efficiency as high as 86.06%, which was significantly higher compared to 57% encapsulation efficiency reported in the previous study using PLGA NPs for siRNA delivery [34]. Zeta potential measurement provides vital information about the surface charge present on the nanoparticles which is measured in the presence and absence of siRNA at physiological pH. In this study, zeta potential of the blank mPEGPLGA-PLL NPs decreased from +11.4 to −0.33 mV when the siRNA was encapsulated in the core of NPs, which might be due to the neutralization effect of the negative charge of siRNA on the positive charge of the pure copolymers. Perhaps, the decrease in zeta potential was also partly attributed to some negatively charged siRNA adsorbed onto the NPs surface. The particle size of NPs loaded with siRNA increased because the siRNA molecules were encapsulated in the inner water phase of mPEG-PLGA-PLL NPs.

The degradation time of PLGA can be altered from days to years by varying the molecular weight, the lactic acid to glycolic acid ratio in copolymer, or the structure of nanospheres. In our study, biodegradable NPs fabricated using mPEG-PLGA-PLL triblock copolymers showed a longer-lasting sustained siRNA release. This finding represents an important advantage for future *in vivo* applications of mPEG-PLGA-PLL NPs-based gene delivery systems.

Our results demonstrated that mPEG-PLGA-PLL NPs had much higher intracellular siRNA delivery efficiencies than siRNA alone and Lipo2000-siRNA complexes. The significantly enhanced delivery of the siRNA by mPEG-PLGA-PLL NPs might be due to the presence of PLL and mPEG or the small size of NPs. PLL is one of the most used cationic polymers for gene delivery [35], and positively charged amine groups of PLL could make electrostatic interactions with negatively charged phosphate groups of the nucleic acids. Therefore, we speculate that the PLL may enhance the encapsulation efficiency and stability of NPs because they tightly bind siRNA in the inner water phase. The presence of a neutral and hydrophilic mPEG shell layer could also effectively enhance gene delivery by promoting the biocompatibility of NPs [36]. A previous study showed that NPs that are 100–200 nm in size offer the best properties for cellular uptake [37]. Therefore, the small size of NPs in our present study makes them more suitable for enhanced siRNA delivery via the endocytotic pathway. The silencing efficiency measured by a FACSCalibur flow cytometry and quantitative real-time RT-PCR showed that mPEG-PLGA-PLL NPs loading siRNA efficiently suppressed GFP expression within the lung cancer cells as compared with Lipofectamine, which was consistent with the high cellular uptake of NPs loading siRNA.

Lipofectamine was the most commonly used and commercially available cationic lipid transfection agent. However, the results of our study showed that Lipofectamine treatment resulted in only 25% inhibition at the protein expression level. The reasons why Lipofectamine-siRNA complexes produced only a slight inhibition of GFP were as follows: in our study, lung cancer SPC-A1-GFP cell line stably expressing GFP was used for analysis of gene silencing. This type of cell line was obtained through infection with the virus bearing the GFP sequence. SPC-A1-GFP cell line showed a very strong GFP expression, therefore, it was very difficult for the Lipofectamine-siRNA complexes to efficiently inhibit GFP expression. Nimesh *et al*. [5] reported that gene silencing efficiency of lipofectamine-siRNA complexes was higher in their studies. We found that the cells used in their studies were co-transfected with GFP plasmid and GFP-siRNA using Lipofectamine according to manufacturer’s protocol. No GFP accumulation existed in the cells treated by this method. However, it was completely different from the transfection method used in our study (SPC-A1-cells was firstly transfected with virus bearing the GFP sequence, and then was transfected again with lipofectamine-siRNA complexes).

To further establish the gene knockdown efficiency at the mRNA level, a RT-PCR analysis of SPC-A1-GFP cells was also carried out in our study. The results showed that the inhibition effect of Lipofectamine-siRNA in SPC-A1-GFP cells was 43.72%, indicating that Lipofectamine treatment was efficient. Whereas even though lipofectamine treatment could efficiently knock down the GFP gene expression at the mRNA level, a certain amount of GFP accumulation still existed in SPC-A1-GFP cells. Therefore, after 48 h of transfection, Lipofectamine treatment resulted in only 25% inhibition at the protein expression level.

Low cytotoxicity and high gene transfection efficiency are critical issues in designing current non-viral siRNA delivery vectors. One of the major advantages of mPEG-PLGA-PLL NPs is that the siRNA is delivered to the cells without hampering the metabolic activity of cells as revealed by the results of MTT assay. The mPEG-PLGA-PLL NPs appear to be a safe gene carrier as evident from their low cytotoxicity.

## 3. Experimental Section

### 3.1. Materials

Monomethoxypoly(ethylene glycol) (mPEG) with a molecular weight of 2 kDa was obtained from Aldrich. L-lactide and glycolide were purchased from Beijing Yuanshengrong Company (Beijing, China). Nɛ-carbobenzyloxy-L-lysine N-carboxyanhydride (Nɛ-CBZ-L-lysine NCA) was purchased from Shanghai Yuanju Biotechnology Company (Shanghai, China).

The active anti-GFP siRNA was synthesized by Ruibo Biotechnology Company (Guangzhou, China) and Cy3 conjugated at the 5' end. The sense and antisense sequences were as follows: 5'-GCU GAC CCU GAA GUU CAU CUG dTdT -3' and 5'-CAG AUG AAC UUC AGG GUC AGC dTdT -3', respectively. In siRNA transfection and gene silencing experiments, Lipofectamine™ 2000 (Invitrogen) was used as a positive control according to the manufacturer’s protocol.

### 3.2. Preparation of mPEG -PLGA-PLL NPs Loading siRNA

**Scheme 1:**
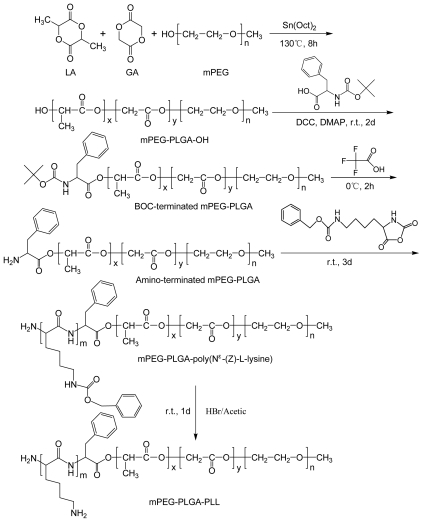
Synthesis of mPEG-PLGA-PLL triblock copolymers.

The mPEG-PLGA-PLL triblock copolymers was synthesized in four steps as shown in Scheme 1: (1) to prepare diblock copolymer mPEG-PLGA-OH by ROP (ring-opening polymerization) of l-lactide and glycolide in the presence of mPEG with stannous octanoate as catalyst; (2) to convert its end-group -OH into -NH_2,_ *i.e*., to prepare mPEG-PLGA-NH_2_. The experimental details were described by Deng *et al*. [38]; (3) to carry out ROP of Nɛ-CBZ-l-lysine NCA in the presence of mPEG-PLGA-NH_2_ as macroinitiator. Purified mPEG-PLGA-PZLL was obtained under vacuum at 40 °C for 24 h; and (4) the final mPEG-PLGA-PLL triblock copolymers were synthesized by deprotecting the copolymer mPEG-PLGA-PZLL in HBr/HAc solution.

Gel permeation chromatography (GPC) measurement was conducted on a Waters 410 GPC with tetrahydrofuran as eluent (flow rate: 1 mL/min, at 35 °C). The molecular weight of mPEG-PLGA-PLL triblock copolymer was calibrated against polystyrene standards.

The degradation behavior of mPEG-PLGA-PLL triblock copolymer was evaluated by the lactate generation. The determination method used in the present study was the same as that used previously to determine the lactate [39]. mPEG-PLGA-PLL samples (1 mL), enclosed in dialysis bags (cellulose membrane, mw cut-off 5 kDa, Sigma-Aldrich, Co., St. Louis, MO, USA), were incubated in 30 mL phosphate buffer saline (PBS, pH 7.4) at 37 °C under mild agitation in a water bath. For lactate assay, 500 μL samples were withdrawn from the incubation medium at predetermined time intervals. The samples were replaced by equal volume of fresh PBS. The lactate formed during mPEG-PLGA-PLL hydrolysis was determined by an enzymatic method based on the quantitative oxidation of lactate to pyruvate in the presence of lactate dehydrogenase (Lactate diagnostic Kit 826, Sigma). The samples were first saponified with an equal volume of a 0.1 M NaOH solution for 30 min at room temperature to hydrolyze lactic acid oligomers that form in solution and thus evade determination. After neutralization with a 0.1 M HCl solution, the L-lactate concentration was measured using the enzymatic assay. The absorbance of the samples was measured at 340 nm.

We prepared mPEG-PLGA-PLL NPs containing siRNA using a w/o/w double emulsion method. Briefly, 50 μL of DEPC-treated water (W_1_) containing 13.3 μg of siRNA were emulsified with 400 μL of CH_2_Cl_2_ (O) containing 8 mg of mPEG-PLGA-PLL with an ultrasonic processor (400 W 10 × 10 s) (JY92-II ultrasonic processor, Ningbo Scientz Biotechnology Co., Ltd., Ningbo, China). Then, the resulting W_1_/O emulsion was poured into 4.4 mL of DEPC-treated water with 1% Pluronic F-68 (W_2_), and the mixture was homogenized by ultrasound (400 W 10 × 10 s). The resulting W_1_/O/W_2_ emulsion was stirred gently at room temperature for 3 h to evaporate the organic solvent. The NPs were passed through a 0.45-μm sieved to remove large particles and then sedimented by centrifugation at 14000 rpm for 30 min. The mPEG-PLGA-PLL NPs containing siRNA were collected by centrifugation, rinsed with distilled water three times, and then lyophilized.

### 3.3. Physicochemical Characterization of NPs Loading siRNA

NPs were observed using an S-4800 field emission scanning electron microscope (SEM) (Hitachi, Tokyo, Japan). The samples were coated with 25-nm-thick gold using a quick carbon coater (SC-701, Sanyu Electronics, Tokyo, Japan). The NPs containing siRNA were collected by centrifugation, rinsed with distilled water three times to remove free siRNA, and then suspended in 0.1 M PBS (pH 7.4). The diameter and zeta potential of the final NPs in 0.1 M PBS (pH 7.4) were measured by the Nicomp 380 ZLS Particle Size and Zeta Potential Analyzer (PSS Nicomp, Santa Barbara, CA, USA).

The siRNA encapsulation efficiency in NPs was determined by high performance liquid chromatography (HPLC). The method used in the present study was the same as that used previously to measure the siRNA encapsulation efficiency [40]. In brief, NPs (4 mg) were dissolved in 0.1 mL of acetonitrile, and then 0.4 mL of phosphate buffer (pH 6.0) was added. After vigorous shaking for 2 h, the supernatants collected by centrifugation (14000 rpm, 30 min) were analyzed using a model 1200 series HPLC (Agilent Technologies, Santa Clara, CA USA) with an ultraviolet (UV) detector as follows: a 4.6 mmΦ × 30 cm TSK-Gel SuperSW3000 column (Tosoh Biosciences, Inc. Stuttgart, Germany); mobile phases, (A) 0.1 M triethylamine-acetic acid solution, (B) acetonitrile; flow rate, 1.0 mL/min; wave length, 260 nm; injection volume, 20 μL. The ratio (%) of the measured, to the formulated amount of siRNA was defined as the encapsulation efficiency of siRNA in NPs.

In the *in vitro* release experiment, NPs (20 mg) were suspended in 2.0 mL of 0.1 M phosphate buffer (pH 7.4), and stirred with a rotator at 37 °C. The NPs and supernatants were periodically collected by centrifugation at 14,000 rpm for 30 min. The amount of residual siRNA in NPs was determined using the same analytical method of encapsulation efficiency. The amount of released siRNA was also determined by HPLC. The released siRNA (%) from NPs was calculated as a ratio (%) of the released amount, to the total amount of detected siRNA (μg).

### 3.4. Gel Retardation Assay

The binding of siRNA with mPEG-PLGA-PLL was determined by 4% agarose (low melting point) gel electrophoresis. Three different weight ratios of mPEG-PLGA-PLL/siRNA nanocomplexes were loaded (20 μL of the sample containing 0.2 μg of siRNA). A 1:6 dilution of loading dye was added to each well and electrophoresis was carried out at a constant voltage of 55 V, for 1 h in TBE buffer (4.45 mM Tris–base, 1 mM sodium EDTA, 4.45 mM boric acid, pH 8.3) containing 0.5 μg/mL ethidium bromide. The siRNA bands were then visualized under a UV transilluminator at a wavelength of 365 nm.

### 3.5. Cell Culture

Human lung cancer SPC-A1 cell line and SPC-A1-GFP cell line stably expressing green fluorescent protein were kindly donated by Cancer Institute of Renji Hospital Affiliated to Shanghai Jiaotong University School of Medicine (Shanghai, China). Both cell lines were cultivated in Dulbecco’s modified Eagle’s medium (DMEM) (Hyclone, Logan, UT, USA) supplemented with 10% fetal bovine serum (FBS) at 37 °C and 5% CO_2_ atmosphere.

### 3.6. Celluar Uptake Studies

SPC-A1-GFP cells were seeded in 24-well plates at a density of 1 × 10^5^ cells per well, and incubated in 500 μL DMEM with 10% FBS for 24 h. Then, the medium was removed by aspiration, and the cells were washed twice with phosphate buffered saline. Lipofectamine-siRNA complexes were prepared as follows: First, dilute 15 μL siRNA in 35 μL of FBS-free DMEM. Mix gently and incubate for 5 minutes at room temperature. Second, dilute 2 μL Lipofectamine™ 2000 in 48 μL of FBS-free DMEM. Mix gently and incubate for 5 minutes at room temperature. Last, after the 5-minute incubation, combine the diluted siRNA with the diluted Lipofectamine™ 2000. Mix gently and incubate for 20 minutes at room temperature to allow complex formation to occur. siRNA alone, Lipofectamine-siRNA complexes or nanoparticles loading siRNA (equivalent with 0.8 μg siRNA each well) and a certain amount of FBS-free DMEM (siRNA alone group: 485 μL, Lipofectamine-siRNA complexes group: 400 μL, siRNA-loaded nanoparticles group: 310 μL) were added to the 24-well plates ensuring that the final volume per well was 500 μL. After 12 h of culture, the transfection medium was removed and the cells were washed three times with phosphate buffer saline. Then, the cells were fixed at room temperature for 15 min with 4% paraformaldehyde, washed with PBS and mounted with Slow Fade® reagent (Moleculer probes Inc., Eugene, OR, USA) containing the nuclear stain Hoechst 33258 (Dojindo Lab., Kumamoto, Japan). Intracellular siRNA uptake and localization were imaged using an Olympus IX-51 fluorescence microscopy (Olympus Optical Company, Ltd., Tokyo, Japan; exposure time: 0.5 seconds and camera aperture: 0.3) and a confocal laser scanning microscopy (LSM 510 META, Carl Zeiss Inc., Jena, Germany) equipped with argon (488 nm) and HeNe (543 nm) lasers. Intracellular fluorescence was determined using a fluorescence-activated cell sorter (FACS) Calibur flow cytometer (Becton Dickinson, CA, USA) and the data were analyzed by Win MDI softwares. All experiments were carried out in triplicate.

### 3.7. Analysis of Gene Silencing

SPC-A1 and SPC-A1-GFP lung cancer cells were seeded in 24-well plates at a density of 1 × 10^5^ cells per well, and incubated in 500 μL DMEM with 10% FBS for 24 h. SPC-A1 lung cancer cells that were not treated with siRNA were used as a negative control. siRNA alone, Lipofectamine-siRNA complexes or nanoparticles loading siRNA (equivalent with 0.8 μg siRNA each well) was added to stable GFP expressing SPC-A1 cell cultures and a certain amount of FBS-free DMEM (siRNA alone group: 485 μL, Lipofectamine-siRNA complexes group: 400 μL, siRNA-loaded nanoparticles group: 310 μL) was also added to the 24-well plates ensuring that the final volume per well was 500 μL. After 12 h of incubation, the transfection medium was replaced with 500 μL of fresh culture media containing 10% FBS and the cells were incubated at 37 °C for 48 h in a humidified 5% CO_2_ atmosphere. The efficiency of GFP silencing was visualized under an Olympus IX-51 fluorescence microscope (exposure time: 0.05 seconds and camera aperture: 0.3) and measured by FACS analysis.

After 48 h of transfection, inhibition of the GFP gene mRNA expression was also evaluated by using quantitative real-time reverse transcription-polymerase chain reaction (RT-PCR). SPC-A1-GFP cells were treated with various siRNA formulations as above-described in transfection and gene silencing experiments. The cells were harvested, lysed, and total RNA was extracted from respective samples using Trizol Reagent Kit (Invitrogen, Karlsruhe, Germany), according to the manufacturer's protocol. Additionally, SPC-A1-GFP cells that were not treated with siRNA were used as a control, and also collected to measure the mRNA levels. Then, reverse transcription to synthesize cDNA was achieved using First Strand cDNA Synthesis Kit (GeneCopoeia Inc, Rockville, MD, USA). RT-PCR was performed with the extracted total RNA by using SYBR® Premix Ex Taq™ (Perfect. Real Time) kit (TaKaTa, Tokyo, Japan). The PCR primers used to detect GFP were: (forward) 5′-TGGCGATGGCCCTGTCCTTT -3′; (reverse) 5′-TGCCATGTGTAATCCCAGCAGCT -3′. Quantification, using the 2^−ΔΔCT^ analytical method, was performed in triplicate with GAPDH as the internal standardization.

### 3.8. Cytotoxicity

The cell viability in the different treatment groups and control group was evaluated by MTT method. SPC-A1-GFP cells were seeded onto 96-well plates at a density of 1 × 10^4^ cells/well and incubated for 24 h to allow for cell adherence. The cells were treated as described in the transfection experiment with siRNA alone, Lipofectamine-siRNA complexes or nanoparticles loading siRNA. After 48 h of transfection, 20 μL of 5 mg/mL MTT [3-(4,5-dimethylthiazol-2-yl)-2,5-diphenyltetrazolium bromide] solution was added to each well, and cells was incubated for 4 h at 37 °C. Then, the MTT containing medium was aspirated, and the formazan crystals formed by the living cells were dissolved in 150 μL DMSO. The absorbance was measured spectrophotometrically in an ELISA plate reader at 570 nm. Untreated cells were taken as control with 100% viability and cells without the addition of MTT were used as blank to calibrate the spectrophotometer to zero absorbance. The relative cell viability (%) compared to control cells was calculated by [abs]_sample_/[abs]_control_ × 100.

### 3.9. Statistical Analysis

Data were expressed as the means and standard deviation (mean ± SD). Independent samples t-test was used to determine the significance of the difference between two groups. Analysis of variance (ANOVA) was used to test for significance by multiple comparisons. Differences were considered significant at *P* < 0.05. Statistical analyses were performed with a software package (SPSS, version 13.0; SPSS, Chicago, IL, USA).

## 4. Conclusions

In conclusion, development of safe and efficient non-viral vectors has received tremendous attention in the hope of finding a substitute for viral vectors. The novel biodegradable mPEG-PLGA-PLL NPs could successfully transfer siRNA into lung cancer cells, and produced higher gene inhibition efficiency than commercially available transfecting agent Lipofectamine, while showing no cytotoxicity. The results of this study suggest that biodegradable NPs of mPEG-PLGA-PLL triblock copolymers can be potentially applied as novel non-viral vectors for improving siRNA delivery and gene silencing. Our future study will focus on the surface-modification of mPEG-PLGA-PLL triblock copolymers with various kinds of ligands to achieve active targeting to tissues and cells, thereby building the “perfect vector” for systemic gene therapy against cancer.
